# Transarterial therapy combined with bevacizumab plus immune checkpoint inhibitors as a neoadjuvant therapy for locally advanced HCC

**DOI:** 10.3389/fimmu.2024.1469302

**Published:** 2024-12-23

**Authors:** Zhenyun Yang, Qianyu Wang, Li Hu, Xiaoxian Sima, Juncheng Wang, Dandan Hu, Zhongguo Zhou, Minshan Chen, Yaojun Zhang, Yizhen Fu

**Affiliations:** ^1^ Department of Liver Surgery, Sun Yat-sen University Cancer Center, Guangzhou, Guangdong, China; ^2^ Collaborative Innovation Center for Cancer Medicine, State Key Laboratory of Oncology in South China, Sun Yat-Sen University Cancer Center, Guangzhou, Guangdong, China; ^3^ Guangdong Provincial Clinical Research Center for Cancer, Sun Yat-Sen University Cancer Center, Guangzhou, Guangdong, China; ^4^ Key Laboratory of Carcinogenesis and Translational Research (Ministry of Education/Beijing), Laboratory of Molecular Oncology, Peking University Cancer Hospital and Institute, Beijing, China; ^5^ Department of Medical Oncology, Sun Yat-Sen University Cancer Center, Guangzhou, Guangdong, China

**Keywords:** hepatocellular carcinoma, neoadjuvant therapy, transarterial therapy, bevacizumab, immune checkpoint inhibitors

## Abstract

**Background:**

Transarterial therapy (TAT), bevacizumab (Bev), and immune checkpoint inhibitors (ICIs) have individually exhibited efficacy in treating advanced-stage hepatocellular carcinoma (HCC). This study aimed to assess the efficacy and safety of the combination of these three treatments as a neoadjuvant modality in patients with locally advanced HCC.

**Methods:**

The primary endpoint is overall survival (OS). The second endpoint is progression free survival (PFS), objective response rate (ORR), pathological response rate and safety.

**Results:**

A total of 54 patients received standard systemic therapy comprising Bev combined with ICIs (Bev-ICIs group), 113 patients received direct surgery (Surgery group), and 273 patients received neoadjuvant therapy of TAT combined Bev plus ICIs, among which 79 patients (28.9%) underwent surgical resection after successful tumor downstaging (Neo-surgery group) while the remaining 194 patients (71.1%) received maintenance systemic therapies (Neo-maintenance group). Neoadjuvant following surgery demonstrated a prolonged OS in contrast to direct surgery, with a median OS time not reached in the Neo-surgery group and 30.6 (95% CI: 26.4-34.7) months in the Surgery group (hazard ratio (HR)=0.29, P=0.0058). The median PFS time in the Neo-surgery and Surgery groups stood at 19.2 (95% CI: 16.1-22.2) and 6.3 (95% CI:4.7-8) months, respectively (HR=0.25, P<0.0001). In patients failed to receiving resection after neoadjuvant therapy, the median OS was 22.8 (95% CI: 22.3-23.1) months, whereas that for the standard care population was 19.7 (95% CI: 15.9-24) month (HR=0.53, P=0.023). The median PFS time in Neo-maintenance group and Bev-ICIs groups was 11.2 (95% CI: 10.4-11.9) and 6.4 (95% CI: 4.4-8.5) months (HR=0.60, P=0.024). The ORR and disease control rate (DCR) across all patients received TAT-Bev-ICIs were 38.8% and 89.4%, respectively. Additionally, the pathological complete response (pCR) rate and the major pathological response (MPR) rate were 22.8% and 48.1% in the Neo-surgery group. As for safety, neoadjuvant therapy did not increase the perioperative complications when compared to direct surgery, and demonstrated similar incidences and severity of AEs when compared to the standard systemic therapy.

**Conclusion:**

The triple therapy regimen comprising TAT-Bev-ICIs emerged as a promising therapeutic strategy for locally advanced hepatocellular carcinoma (HCC) as a neoadjuvant intervention.

## Introduction

Liver malignancy, a prevalent form of solid tumors, ranks as the third most frequent cause of cancer-related mortality globally (1). Hepatocellular carcinoma (HCC) emerges as the predominant subtype of primary liver neoplasms worldwide, exhibiting a steadily escalating incidence rate on a global scale (2). Regrettably, about 40% patients with HCC were in locally advanced stage when diagnosed, characterized by the presence of multiple tumor nodules or macrovascular invasion, which precludes them from being suitable candidates for surgical resection in accordance with the guidelines established by the European Association for the Study of the Liver and the American Association for the Study of Liver Diseases (3–5). Nevertheless, in Asia, patients afflicted with locally advanced HCC, who fulfill specific criteria, for instance complete excision of tumors confined to a single lobe, remain viable candidates for surgical resection (6–9). Unfortunately, even surgical resection and diligent postoperative care, a majority of patient experience recurrence of HCC following primary surgery (10, 11). The emergence of neoadjuvant therapy has demonstrated promise in affording additional surgical prospects for patients with unresectable lesions (12–14). Although some studies investigated neoadjuvant therapy in HCC patients, the imperative remains to innovate novel and dependable therapeutic modalities to mitigate recurrence and enhance the prognosis of those with locally advanced HCC.

In recent years, considerable attention has been directed towards the confluence of immune checkpoint inhibitors (ICIs)and antiangiogenesis as a therapeutic strategy for advanced HCC (15). Notably, the IMbrave150 trial demonstrated that the combination of atezolizumab (a programmed death ligand 1 (anti-PDL1) inhibitor) with bevacizumab (a vascular endothelial growth factor receptor (VEGFR) inhibitor) yielded enhanced progression-free survival (PFS) and overall survival (OS) outcomes in patients with unresectable HCC, in comparison to treatment with sorafenib alone. The ORIENT-32 trial elucidated that the combination of sintilimab (a programmed death 1 (PD1) inhibitor) with bevacizumab yielded notable improvements in OS, PFS, objective response rate (ORR), and quality of life, surpassing outcomes observed with sorafenib (16, 17). Meanwhile, transarterial therapy (TAT) including transarterial chemoembolization (TACE) and hepatic arterial infusion chemotherapy (HAIC) remains the main choice for patients with intermediate to advanced HCC (18). Extensive studies suggest that combining TAT with immune checkpoint inhibitors (ICIs) may offer superior survival advantages compared to monotherapy (19–22). Consequently, the integration of transarterial therapy with bevacizumab and ICIs (TAT-Bev-ICIs) emerges as a potentially efficacious regimen for the management of locally advanced HCC.

At present, there exists a paucity of data concerning neoadjuvant modalities for HCC patients (23). Despite the recent strides made in combination therapies for advanced HCC, the optimal integration of these treatments into the management schema for resectable and potentially resectable HCC remains elusive. In the framework of this longitudinal, retrospective, real-world investigation, our objective is to assess the efficacy and safety profile of TAT-Bev-ICIs as neoadjuvant therapy in patients with locally advanced HCC.

## Materials and methods

### Patients and data collection

**Figure 1:**
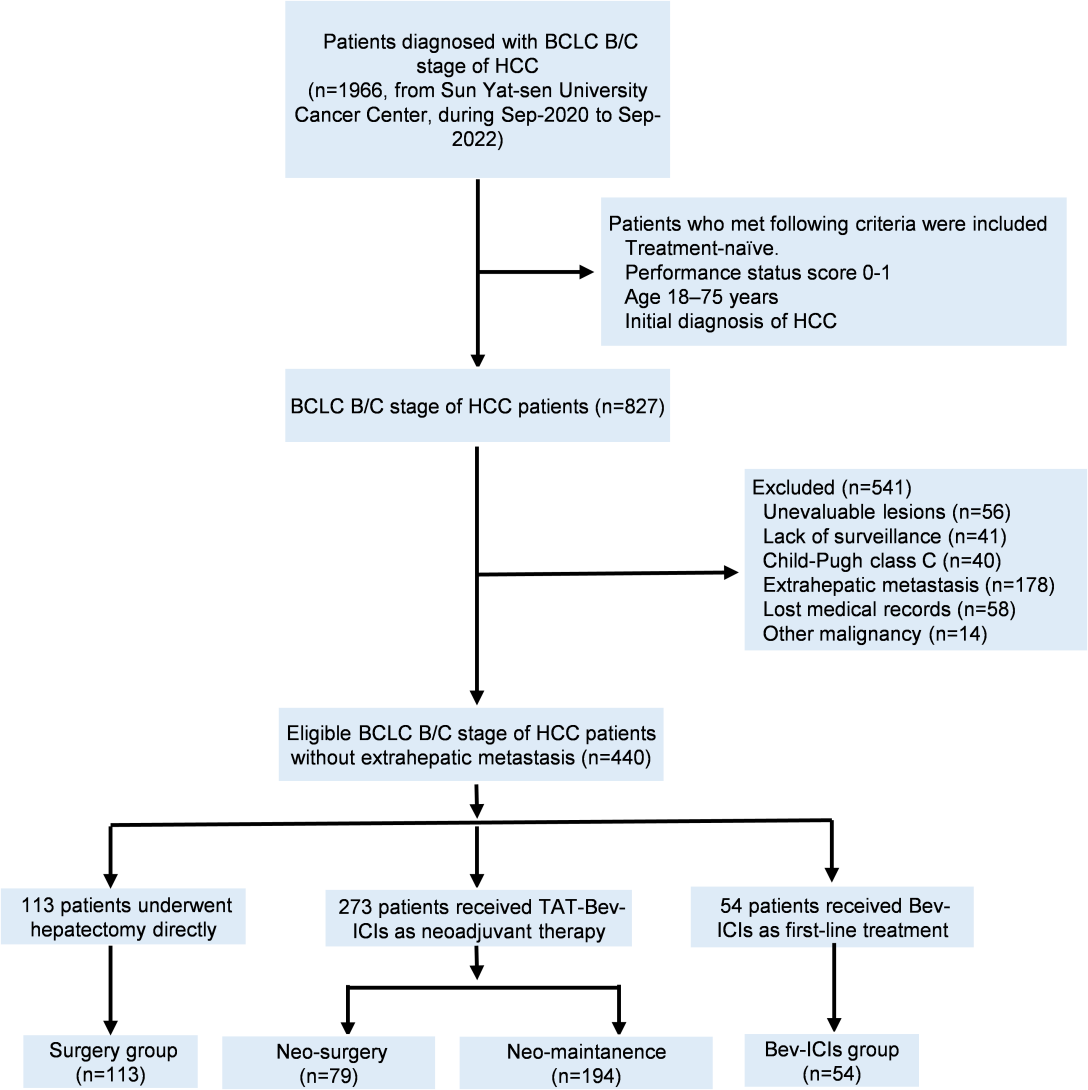
The flowchart of patients. BCLC Barcelona clinic liver cancer; HCC, hepatocellular carcinoma; TAT, transarterial therapy; Bev, bevacizumab; ICIs, immune checkpoint inhibitors.

Between September 2020 and September 2022, 1966 consecutive patients diagnosed with BCLC B/C stage of HCC received surgical resection directly or Bev plus ICIs at Sun Yat-sen University Cancer Center were retrospectively reviewed. The inclusion criteria were as follows: (1) Treatment-naïve; (2) Performance status score 0-1; (3) Age 18–75 years; (4) Initial diagnosis of HCC; (5) No history of other malignancies. The exclusion criteria were as follows: (1) Unevaluable lesions; (2) Lack of surveillance; (3) Child-Pugh class C; (4) Extrahepatic metastasis; (5) Lost medical records and (6) Other malignancy. Finally, a total of 440 HCC patients were enrolled in the study. Among them, 54 patients received Bev combined with ICIs (Bev-ICIs group), 113 patients received surgical resection directly (Surgery group), and 273 patients received neoadjuvant therapy of TAT combined Bev plus ICIs (Neo group). The detailed inclusion flowchart was shown in **Figure 1**.

### Treatments

Patients allocated to the Bev-ICIs group were administered either 1200 mg of atezolizumab or 200 mg of sintilimab in combination with 15 mg per kilogram of body weight of bevacizumab intravenously at three-week intervals. Surveillance assessments were conducted every six weeks (2 cycles of treatments), and treatment was continued until disease progression or intolerable toxicity.

Patients in TAT-Bev-ICIs group received the transarterial therapy every 3 weeks, concomitant with bevacizumab and immunotherapy upon treatment commencement. The transarterial therapy encompassed transcatheter arterial chemoembolization (TACE) and hepatic arterial infusion chemotherapy (HAIC) utilizing a regimen of 5−fluorouracil plus oxaliplatin (FOLFOX), with treatment protocols informed by previous study (24). The interval between HAIC and TACE was 3 weeks, while the interim period between transarterial therapy and immunotherapy was less than one day. Efficacy assessments were conducted at six-week intervals, with the total treatment regimen determined by a multidisciplinary team (MDT) in accordance with tumor response. Typically, patients achieving tumor downstaging would discontinue adjuvant therapy and proceed to liver resection when residual liver volume was sufficient, while those with stable disease would undergo up to six cycles of transarterial therapy before transitioning to Bev-ICIs maintenance therapy. Patients experiencing progressed disease or intolerable toxicity would be guided towards appropriate second-line treatments by the MDT committee.

### Outcomes

The primary endpoint encompasses overall survival (OS), delineated as the span from treatment initiation to cancer-related death or the latest follow-up. The second endpoint include progression-free survival (PFS), objective response rate (ORR), pathological response rate and safety. PFS is characterized by the duration from treatment commencement to disease progression, mortality, or the latest follow-up. Response Evaluation Criteria in Solid Tumors (RECIST, version 1.1) and modified RECIST1.1 (mRECIST) criteria were employed for tumor response evaluations (25, 26). ORR represents the proportion of patients with confirmed complete response (CR) or partial response (PR), while disease control rate (DCR) encompasses objective response coupled with stable disease (SD). MPR was characterized by a range of 90%–99% tumor necrosis within the resected tissue. pCR was delineated as the absence of residual cancer cells in the resected tissue (14, 27). Both imaging and pathology were identified by two specialists in their respective fields. Adverse events (AEs) were appraised following CTCAE version 5.0 guidelines.

### Statistical analysis

Continuous parametric data were are presented as median (IQR) and were analyzed with the Mann-Whitney U test. The Mann-Whitney U test is a non-parametric statistical method used to compare whether two independent samples come from the same distribution. The analysis of categorical data typically involves comparing the frequencies of events across different groups. The Pearson chi-square test and Fisher’s exact test are two commonly used statistical methods. The Pearson chi-square test is suitable for large samples, while Fisher’s exact test is appropriate for small samples or when the data do not meet the expected frequency conditions for the chi-square test. Kaplan-Meier curve is a nonparametric statistical method used to describe changes in survival data over time and is commonly used in medical studies to assess OS and PFS. The log-rank test is often used to compare whether there are significant differences in survival distributions between two groups. To identify independent risk factors for OS and PFS, Cox regression analysis was employed. Initially, univariate regression was conducted, and variables with P-values below 0.1, as well as those that might influence tumor progression or survival based on clinical insight, were included in the multivariate analysis, which followed the forward conditional method. Statistical significance was designated for P-values below 0.05. All statistical analyses were carried out using Statistical Product and Service Solutions (SPSS, version 25.0, SPSS Inc., Chicago, USA) and R software (version 4.3.0, R Foundation, Vienna, Austria).

## Results

### Characteristics of patients

Between September 2020 and September 2022, a cohort comprising 440 locally advanced HCC patients was enrolled in this study. Among them, 54 patients received standard systemic therapy of Bev-ICIs (Bev-ICIs group), 113 patients underwent direct hepatectomy (Surgery-group), and 273 patients received TAT-Bev-ICIs as neoadjuvant therapy. Within the TAT-Bev-ICIs cohort, 79 patients (28.9%) achieved tumor downstaging and subsequently underwent surgery (Neo-surgery group), whereas the remaining 194 patients (71.1%) received continuous Bev-ICIs treatment after completing up to 6 cycles the neoadjuvant therapy (Neo-maintenance). **Figure 1** illustrates the enrollment process. The baseline characteristics of patients in the TAT-Bev-ICIs group are delineated in **Supplementary Table S1.**

### Comparison among surgical patients

We first conducted a comparative analysis between the Neo-surgery group and the Surgery group. Notably, these was no statistically significant difference in baseline characteristics between these two cohorts before the initiation of neoadjuvant treatment and hepatectomy (**Table 1**). In the Neo-surgery group, the median tumor diameter measured 8.3 cm, the majority of patients (73.4%) presented with multiple tumors, and macrovascular invasion was observed in 34 (43%) patients. Within the Surgery group, the median tumor diameter was 7.7 cm, the majority of patients presented with multiple tumors (69.9%), and macrovascular invasion was observed in 52 (46%) patients. The initial baseline characteristics between the two patient groups before treatments showed no statistically significant differences. Additionally, the characteristics between these two cohorts before surgery were shown in **Supplementary Table S2**. Patients in Neo-surgery group had a smaller tumor size than Surgery group due to neoadjuvant therapy.

**Table 1:** Baseline characteristics of the 192 patients received surgery before treatment.

Variables	Neoadjuvant- surgery (n=79)	Surgery (n=113)	*P* value
Age, years	54.1 (46.5-60.6)	54.9 (48-61.5)	0.792
Sex			0.394
Male	68 (86.1)	92 (81.4)	
Female	11 (13.9)	21 (18.6)	
Hepatitis infection			0.371
Yes	64 (81)	97 (85.8)	
No	15 (19)	16 (14.2)	
Liver cirrhosis			0.677
Yes	48 (60.8)	72 (63.7)	
No	31 (39.2)	41 (36.3)	
Preoperative blood tests			
ALT, IU/L	34.6 (25-58.6)	33.3 (22.4-48.7)	0.194
AST, IU/L	43.3 (27.9-65.5)	37.6 (25.5-54.3)	0.326
ALB, g/L	44.2 (42.2-47)	43.7 (41.4-46.3)	0.138
TBil, μmol/L	12.8 (9.8-17)	12.8 (9.5-19)	0.652
AFP, ng/mL	290.8 (5.3-6362)	486 (18.2-5116)	0.394
WBC, ×10^9^/L	7.1 (6.1-8.5)	6.9 (5.7-8)	0.164
HGB, g/L	146 (134-159)	149 (140-159)	0.425
PLT, ×10^9^/L	217 (173-318)	216 (158-307)	0.322
PT	11.5 (11-12.1)	11.7 (11.2-12.3)	0.171
Largest tumor size, cm	8.3 (5.7-10.8)	7.7 (5.5-10)	0.153
Tumor number			0.597
Single	21 (26.6)	34 (30.1)	
Multiple	58 (73.4)	79 (69.9)	
Macrovascular invasion			0.683
Yes	34 (43)	52 (46)	
No	45 (57)	61 (54)	
ALBI grade			0.813
I	69 (87.3)	100 (88.5)	
II	10 (12.7)	13 (11.5)	
III	0	0	
BCLC stage			0.683
B	45 (57)	61 (54)	
C	34 (43)	52 (46)	

Data are presented as median (IQR), or n (%).

AST, aspartate transaminase; ALT, alanine transaminase; ALB, albumin; TBIL, total bilirubin; AFP alpha-fetoprotein; WBC, white blood cell; HGB, hemoglobin; PLT platelet count; PT, prothromnine time; ALBI grade, Albumin-Bilirubin grade; BCLC, Baecelona clinic liver cancer.

**Figure 2:**
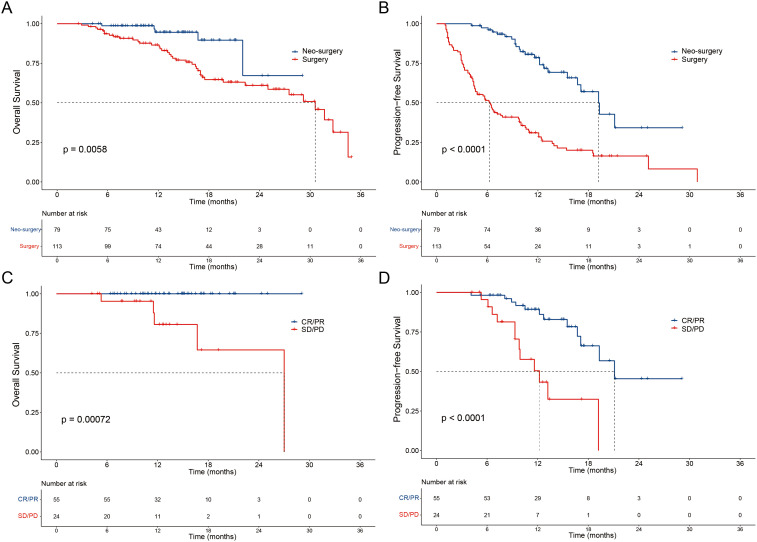
Patient survival was shown by the Kaplan–Meier curves. The OS **(A)** and PFS **(B)** in patients treated with Neo-surgery versus Surgery. The OS **(C)** and PFS **(D)** in patients received Neo-surgery with tumor response (CR/PR vs SD/PD) according to mRECIST criteria. OS, overall survival; PFS, progression-free survival; Neo, neoadjuvant; mRECIST, Modified Response Evaluation Criteria in Solid Tumors; CR, complete response; PR, partial response; SD, stable disease; PD, progressive disease.

Notably, the Neo-surgery group demonstrated a prolonged OS in contrast to the Surgery group, with a median OS time not reached in the Neo-surgery group and 30.6 (95% CI: 26.4-34.7) months in the Surgery group (hazard ratio (HR)=0.29, P=0.0058; **Figure 2A**). Concurrently, the median PFS time in the Neo-surgery and Surgery groups stood at 19.2 (95% CI: 16.1-22.2) and 6.3 (95% CI:4.7-8), respectively (HR=0.25, P<0.0001; **Figure 2B**). In the Neo-surgery group, noteworthy improvements in OS were seen in patients with positive tumor response (CR and PR) in contrast to non-responders (SD and PD) (P = 0.00072; **Figure 2C**). Furthermore, responders (CR and PR) had a prolonged PFS compared to non-responders (SD and PD) (P < 0.0001; **Figure 2D**). Moreover, in the Neo-surgery group, 22.8% patients (18 of 79) had confirmed pCR after resection and 48.1% patients (38 of 79) had confirmed MPR. In addition, the pCR patients had a prolonged PFS compared to non-pCR (P=0.012) and there were no deaths in pCR patients (**Supplementary Figure S1**).

Moreover, operative details of two groups were shown in **Table 2**. In general, Neo-surgery group had more blood loss and more operative time than Surgery group. However, there was no significant difference in postoperative complications between two groups. Meanwhile, patients who received TAT-Bev-ICIs were encompassed within the safety analysis (**Supplementary Table S3**). Predominant AEs of any grade were fever (39.9%), abdominal pain (30.8%), vomiting (23.8%), elevated alanine aminotransferase (ALT) (35.9%), elevated aspartate aminotransferase (AST) (33.7%), and et al.

**Table 2:** Operative details of the 192 patients received liver resection.

Variables	Neo-surgery (n=79)	Surgery (n=113)	*P* value
Blood loss(ml)	300 (200-500)	200(100-375)	0.001
Plasma transfusion, n (%)	7 (8.9)	7(6.2)	0.484
Erythrocyte transfusion, n (%)	10 (12.7)	9 (8)	0.284
Hospital stay(days)	6 (5-6)	5 (5-6)	0.493
Operative time(minutes)	180 (150-210)	132.5 (120-163.8)	<0.0001
Pringle maneuver, n (%)	73 (92.4)	72 (63.7)	<0.0001
Clamping times(minutes)	24.5 (16-34)	25(8.5-40.8)	0.591
Postoperative complications, n (%)	34 (43)	41(35.4)	0.345
Hepatic insufficiency	0	1	
Biliary leakage	10	11	
Pleural effusion	8	10	
Peritoneal encapsulated effusion	10	15	
Ascites	2	4	
Pulmonary infection	1	3	
Wound infection	4	7	
Postoperative bleeding	3	5	
Clavien-Dindo Classification, n(%)			0.584
I	53	76	
II	26	36	
III	0	1	
IV	0	0	

Some patients may have multiple postoperative complications. Data are presented as median (IQR), or n (%).

### Comparison among non-surgical patients

Simultaneously, we compared the Neo-maintenance group with Bev-ICIs group. Overall, the tumor burden was greater in the Neo-maintenance group, evidenced by larger tumor size (8.0cm versus 6.3cm) and more proportion of macrovascular invasion (53.1% versus 38.9%). Comprehensive details are delineated in **Table 3**.

**Table 3:** Baseline characteristics of the 248 patients received neo-maintenance or Bev-ICIs.

Variables	Neo-maintenance	Bev-ICI	*P* value
	(n=194)	(n=54)	
Age, years	57.1 (45-63.9)	56.1 (44.3-60.5)	0.610
Sex			0.102
Male	174 (89.7)	44 (81.5)	
Female	20 (10.3)	10 (18.5)	
Hepatitis infection			0.664
Yes	172 (88.7)	49 (90.7)	
No	22 (11.3)	5 (9.3)	
Liver cirrhosis			0.627
Yes	122 (62.9)	32 (59.3)	
No	72 (37.1)	22 (40.7)	
Preoperative blood tests			
ALT, IU/L	33.2 (25.3-53.6)	26.4 (18.5-50.8)	0.080
AST, IU/L	50.7 (34.1-84.2)	34 (24.8-97.9)	0.125
ALB, g/L	42.3 (39.2-44.9)	42.8 (39.6-45)	0.489
TBil, μmol/L	15 (10.6-19.4)	12.6 (9.5-18.5)	0.155
AFP, ng/mL	191 (9.4-2321)	175.5 (8.3-1550)	0.026
WBC, ×10^9^/L	6.6 (5.4-8.1)	6.2 (4.9-7.1)	0.067
HGB, g/L	142.5 (132.8-150)	140.5 (128.5-156.3)	0.953
PLT, ×10^9^/L	166.5 (141.8-228.5)	167 (126-202.5)	0.455
PT	12.1 (11.3-12.8)	11.9 (11.3-12.5)	0.246
Largest tumor size, cm	8 (5.1-11.4)	6.3 (4.5-8)	0.003
Tumor number			0.880
Single	45 (23.2)	12 (22.2)	
Multiple	149 (76.8)	42 (77.8)	
Macrovascular invasion			0.065
Yes	103 (53.1)	21 (38.9)	
No	91 (46.9)	33 (61.1)	
ALBI grade			0.532
I	129 (66.5)	40 (74.1)	
II	62 (32)	14 (25.9)	
III	3 (1.5)	0	
BCLC stage			0.065
B	103 (53.1)	21 (38.9)	
C	91 (46.9)	33 (61.1)	

Data are presented as median (range), or n (%).

Neo, neoadjuvant; Bev, bevacizumab; ICI, immune checkpoint inhibitors; AST, aspartate transaminase; ALT, alanine transaminase; ALB, albumin; TBIL, total bilirubin; AFP, alpha-fetoprotein; WBC, white blood cell; HGB, hemoglobin; PLT platelet count; PT prothrombin time; ALBI grade, Albumin-Bilirubin grade; BCLC Barcelona clinic liver cancer.

**Figure 3:**
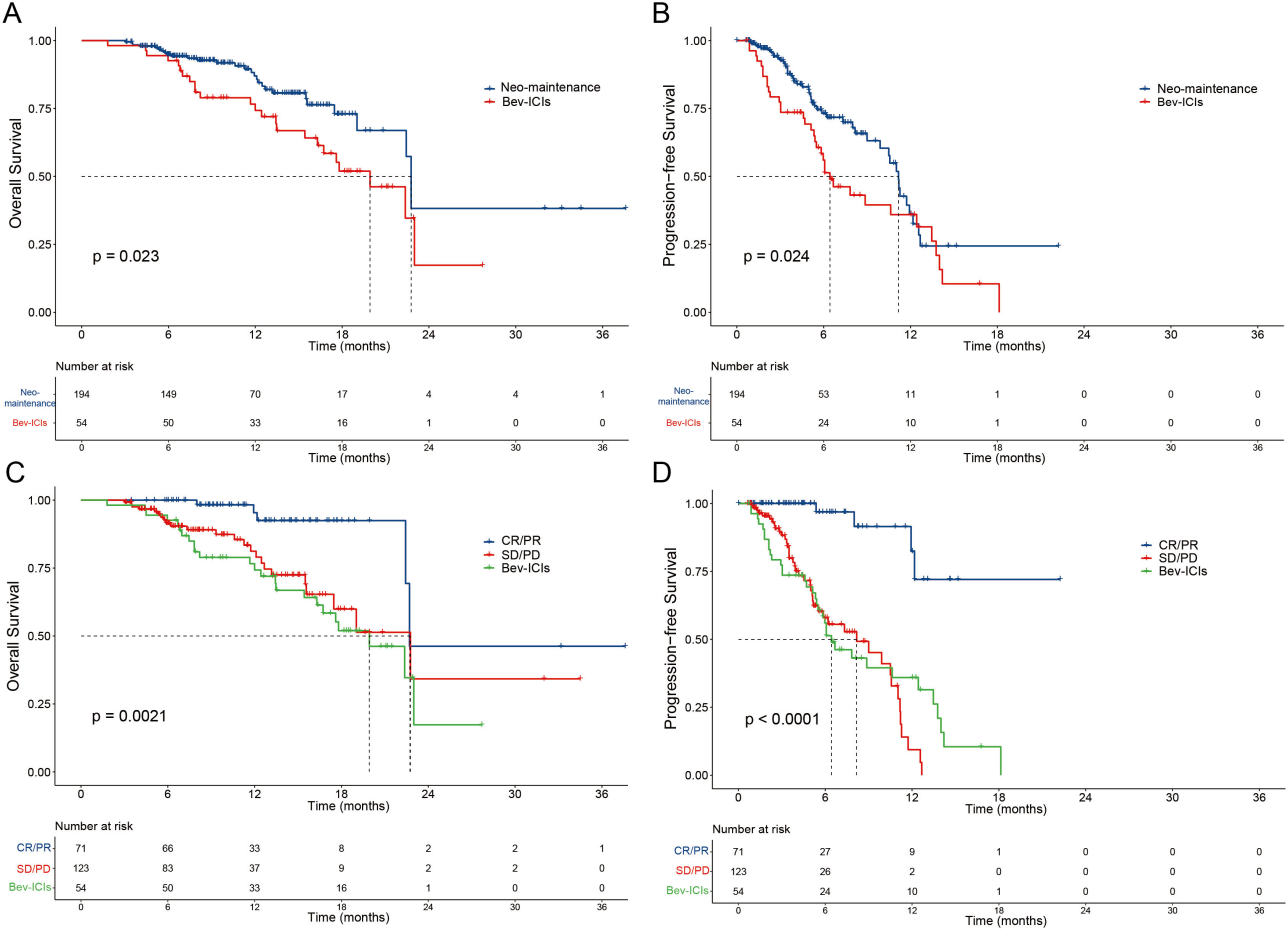
Patient survival was shown by the Kaplan–Meier curves. The OS **(A)** and PFS **(B)** in patients treated with Neo-maintenance versus Bev-ICI. The OS **(C)** and PFS **(D)** in patients received Neo-maintenance with tumor response (CR/PR and SD/PD) versus Bev-ICIs. OS, overall survival; PFS, progression-free survival; Neo, neoadjuvant; Bev, bevacizumab; ICIs, immune checkpoint inhibitors; CR, complete response; PR, partial response; SD, stable disease; PD, progressive disease.

The median OS time was 22.8 (95% CI: 22.3-23.1) months for the Neo-maintenance group and 19.7 (95% CI: 15.9-24) months for the Bev-ICIs group (HR=1.84, P=0.023, **Figure 3A**). The median PFS time was 11.2 (95% CI: 10.4-11.9) months for the Neo-maintenance group and 6.4 (95% CI: 4.4-8.5) months for the Bev-ICIs group (HR=1.63, P=0.024, **Figure 3B**). In the Neo-maintenance group, noteworthy improvements in OS were seen in patients with positive tumor response (CR and PR) in contrast to non-responders (SD and PD) and Bev-ICIs group (P = 0.0021; **Figure 3C**). Furthermore, responders (CR and PR) had a prolonged PFS compared to non-responders (SD and PD) and Bev-ICIs group (P < 0.0001; **Figure 3D**).

The Neo-maintenance group exhibited a higher incidence of adverse events compared to the Bev-ICIs group (**Supplementary Table S4**), mainly resulted from the additional transarterial therapy. To be specific, the frequencies of fever (78 [40.2%] vs 5 [9.3%]; P<0.001), abdominal pain (55 [28.4%] vs 4 [7.4%]; P=0.003), elevated ALT (68 [35.1%] vs 10 [18.5%]; P=0.021), and elevated AST (72 [37.1%] vs 9 [16.7%]; P=0.005) were notably increased in the Neo-maintenance group. For patients enrolled in the TAT-Bev-ICIs cohort, the elevation in serum aminotransferases and total bilirubin reached its zenith on the day subsequent to the conclusion of transarterial therapy, swiftly reverting thereafter to baseline levels. For these patients with abnormal liver function, active measures in liver protection and symptomatic relief were promptly administered. Adjustments in medication dosage or discontinuation of treatment were implemented as deemed necessary. It is noteworthy that there existed no discernible divergence in ALT and AST levels pre- and post-treatment, as observed across both cohorts (**Supplementary Figure S2**). Moreover, all AEs were proficiently handled, and there were no mortalities linked to treatment toxicity documented throughout the follow-up phase.

The tumor response in patients receiving Bev-ICIs therapy is presented in **Supplementary Table S5**. Following RECIST 1.1 criteria, the Neo-maintenance group exhibited superior ORR and DCR compared to the Bev-ICIs group (ORR: 31.9% vs. 14.8%, P = 0.013; DCR: 85.1% vs. 63%, P < 0.0001). Similar findings were observed when employing mRECIST criteria.

### Response rate and predictors of neoadjuvant therapy

The tumor responses among patients undergoing TAT-Bev-ICIs therapy are delineated in **Table 4**. According to RECIST 1.1 criteria, the ORR and DCR across all patients were 38.8% and 89.4%, respectively. When evaluated using mRECIST criteria, these data stood at 46.2% for ORR and 89.4% for DCR. The waterfall plot depicted in **Supplementary Figure S3** illustrates the optimal response of intra-hepatic target lesions, based on RECIST 1.1 criteria.

**Table 4:** Best tumor responses evaluated by RECIST1.1 and mRECIST criteria for patients received TAT-Bev-ICIs.

		RECIST1.1			mRECIST		RECIST1.1	mRECIST
Response	Neo-surgery	Neo-maintenance	*P* value	Neo-surgery	Neo-maintenance	*P* value	All patients	All patients
	(n=79)	(n=194)		(n=79)	(n=194)		(n=273)	(n=273)
CR	12 (15.2%)	2 (1%)	–	19 (24.1%)	7 (3.6%)	–	13 (4.8%)	26 (9.5%)
PR	33 (41.7%)	60 (30.9%)	–	36 (45.6%)	64 (33%)	–	93 (34.1%)	100 (36.6%)
SD	34 (43.1%)	103 (53.1%)	–	24 (30.4%)	94 (48.5%)	–	137 (50.2%)	118 (43.2%)
PD	0	29 (14.9%)	–	0	29 (14.9%)	–	29 (10.6%)	29 (10.6%)
ORR	45 (56.9%)	62 (31.9%)	<0.0001	55 (69.6%)	71 (36.6%)	<0.0001	106 (38.8%)	126 (46.2%)
DCR	79 (100%)	165 (85.1%)	<0.0001	79 (100%)	165 (85.1%)	<0.0001	244 (89.4%)	244 (89.4%)

Neo, neoadjuvant; TAT, transarterial therapy; Bev, bevacizumab; ICIs, immune checkpoint inhibitors; CR, complete response; PR, partial response; SD, stable disease; PD, progressive disease; ORR, objective response rate; DCR, disease control rate.

Then, we conducted univariate and Multivariable Cox regression analyses in patients received TAT-Bev-ICIs. Prognostic indicators of all clinical variables were tested in the Univariate analysis. In terms of OS, Univariate analyses underscored the significance of alpha-fetoprotein (AFP) levels and liver resection downstaging as pivotal risk factors. Similarly, the Univariate analysis for PFS highlighted AFP levels, tumor number, macrovascular invasion, and downstaging liver resection as risk factors. Further elucidation is available in **Supplementary Table S6.** The multivariate Cox proportional analysis delineated AFP levels (P=0.016) and liver resection downstaging (P=0.023) as significant and independent prognostic determinants of OS (**Supplementary Table S6**). Additionally, this analysis identified tumor number (P=0.003), macrovascular invasion (P=0.007), and liver resection downstaging (P<0.0001) as notable and autonomous prognostic factors for PFS (**Supplementary Table S6**).

## Discussion

This longitudinal, real-world, retrospective study stands as the first showcase of neoadjuvant TAT-Bev-ICI’s superiority over Bev-ICIs or Surgery directly in attaining the projected clinical outcome of markedly improved OS, PFS, and ORR among patients diagnosed with locally advanced HCC, all the while upholding a satisfactory and well-tolerated safety profile. The strengths of this present study lay in: (1) the incorporation of a real-world, expansive study cohort, (2) a comparative examination between cohorts who underwent tumor downstaging followed by surgery and those who proceeded directly to surgery, (3) a comparative evaluation between cohorts receiving TAT-Bev-ICIs maintenance and Bev-ICIs, and (4) the thorough documentation of both short- and long-term treatment outcomes for patients with locally advanced HCC undergoing TAT-Bev-ICIs.

In this study, it was observed that within the cohort receiving TAT-Bev-ICIs, 38.8% of patients achieved an ORR as per RECIST 1.1 criteria, while 46.2% attained ORR based on mRECIST criteria. Previous studies have delineated that the combination therapy also displayed encouraging efficacy with minimal safety concerns in the therapeutic regimen for patients with HCC in adjuvant setting. For instance, a study elucidated that the combination of camrelizumab and apatinib resulted in an ORR of 16.7% as per RECIST 1.1 and 33.3% according to mRECIST criteria in advanced HCC patients (28). Furthermore, a phase III randomized clinical trial reported that the combined therapy of lenvatinib with TACE achieved ORRs of 45.9% and 54.1% based on RECIST 1.1 and mRECIST criteria, respectively (29).

Additionally, neoadjuvant therapy presents an opportunity to effectively reduce tumor burden in patients, thereby enhancing surgical outcomes in select cases. Within our study cohort, 79 patients (28.9%) receiving TAT-Bev-ICIs achieved tumor downstaging, facilitating subsequent surgical intervention, while 18 patients (22.8%) attained pCR. A phase II clinical trial observed that 17.6% and 5.9% patients who received camrelizumab plus apatinib reported MPR and pCR, respectively (28). Similarly, in a retrospective study that enrolled 41 HCC patients received TACE and tislelizumab therapy as neoadjuvant therapy, pCR and MPR rates was 31.7% and 43.9% (30). Most of patients enrolled in this study was BCLC stage A, which caused the high rates of pCR and MPR. Furthermore, a randomized clinical trial reported that 12.8% of patients achieved curative surgical resection following the combined regimen of HAIC with sorafenib, with an additional 18.8% attaining pCR (31). Conversely, another study revealed that 15.3% of patients underwent curative surgical resection post-TACE combined with lenvatinib, yielding a pCR rate of 7.7% (29). Notably, these rates of curative surgical resection were comparatively lower than those observed in our study. Based on interventional therapy, it is evident that triple therapy holds promise in reducing tumor burden, fostering greater tumor necrosis, and enhancing the likelihood of downstaging surgery compared with dual therapy. Moreover, it highlights the discernible survival advantages conferred by immunotherapy.

Subsequently, a comparative analysis was undertaken between the Neo-surgery cohort and those undergoing direct surgery. Remarkably, our findings unveiled that the Neo-surgery cohort exhibited prolonged OS and PFS compared to their counterparts undergoing immediate surgical intervention. Preclinical investigations and correlative analyses lend credence to the hypothesis that neoadjuvant therapy leads a localized tumor response and mitigates recurrence by modulating the tumor microenvironment, a phenomenon observed across various therapeutic modalities, including immune-based interventions (32, 33). Numerous synergistic mechanisms contributing to the effectiveness of neoadjuvant therapy in HCC have been elucidated. A recurring observation in these trials, as well as in other disease contexts, is the development of tertiary lymphoid structures (TLS), which act as focal points for T cell memory generation and are linked to enhanced survival (34–36). Significantly, responders exhibited an elevated abundance of TLS and a greater presence of tumor-specific CD4+ and CD8+ T cells (37).

In the TAT-Bev-ICIs cohort, barring those who underwent successful tumor downstaging followed by surgical intervention, a majority of patients (71.1%) failed to undergo resection and instead received maintenance therapy with Bev plus ICIs. The primary reasons contributing to the non-surgical status of these patients are delineated as follows: (1) The extent of tumor regression failed to meet anticipated thresholds, thereby resulting in a classification of SD or even PD upon efficacy assessment. (2) Despite achieving tumor reduction to levels indicative of PR, the residual hepatic volume remains inadequate, rendering the patient unsuitable for surgical intervention. (3) Although tumor regression has occurred, rendering surgical intervention viable according to medical evaluation, patients have either declined surgery or present with contraindications pertaining to anesthesia.

A comparative analysis ensued between the Neo-maintenance cohort and the Bev-ICIs cohort. Although Neo-maintenance cohort had a greater tumor burden, the median OS and PFS were notably extended in the Neo-maintenance cohort compared to the Bev-ICIs cohort. This underscores the criticality of incorporating combination TAT in therapeutic strategies. Despite the accumulation of evidence from various clinical investigations affirming the enhanced efficacy of TAT when combined with angiogenesis inhibitors and immune checkpoint inhibitors (ICIs) in the management of advanced hepatocellular carcinoma (HCC), the precise mechanistic underpinnings remain enigmatic (20, 29, 38–40). The effectiveness of the triple therapeutic approach can be elucidated through the following considerations: (1) Following TAT, the hypoxic milieu within the tumor microenvironment may induce angiogenesis. Bevacizumab, through its targeted inhibition of Vascular Endothelial Growth Factor (VEGF) 1–3, can effectively counteract post-TAT angiogenesis (41). (2) TAT in the context of HCC holds potential for modulating tumor immunity by reshaping the tumor microenvironment (42). Through TAT, tumor cell necrosis triggers the release of tumoral neoantigens, thereby facilitating the recruitment and activation of dendritic cells within the microenvironment. This orchestrated effect can serve to convert an immunosuppressive microenvironment, which is less conducive to ICIs, into an immunosupportive milieu, enhancing the efficacy of systemic therapies (43).

In addition to the clinical efficacy outcomes, the safety profile of TAT-Bev-ICIs in patients with locally advanced HCC warrants consideration. Our study revealed a heightened incidence of elevated liver enzymes associated with TAT-Bev-ICIs administration, potentially come from the direct cytotoxic effects exerted on hepatocytes by TAT. Nevertheless, these adverse events remained predominantly manageable and did not precipitate deterioration in the patients’ underlying hepatic condition. Through the judicious application of adjunctive pharmacotherapy, hepatic function could be ameliorated during the interval between successive cycles of TAT therapy. In addition, we also observed that patients in Neo-surgery group had more blood loss and more operative time than Surgery group. It could be explained that TAT might instigate localized inflammation, precipitating adhesions that consequently escalate the complexity of the surgical procedure (44–46). However, the surgery-related complications were controllable. In summary, TAT-Bev-ICIs emerges as a regimen of both efficacy and safety for the management of locally advanced HCC.

This study also has certain limitations. Firstly, this was a retrospective study conducted on a single-center cohort. In retrospective studies, since data is collected from existing records, there may be selection bias, meaning that the selection of study subjects is not random and may be biased towards certain specific groups or conditions. Single-center studies may lead to sample selection bias because patients from different regions may have different genetic backgrounds, lifestyles, and socioeconomic statuses. Therefore, it is necessary to conduct a prospective, 68multicenter, and randomized controlled trial to substantiate our findings. Secondly, the retrospective design inherently carries the risk of incomplete AE assessment, notwithstanding our diligent scrutiny of medical records, which may underestimate the incidence and severity of AEs and affect the evaluation of drug safety. While we assert the comprehensiveness of AE analysis, the validation of our conclusions mandates a prospective, multicenter, and randomized controlled trial.

## Conclusion

This study serves to delineate the efficacy and safety of the triple therapy regimen comprising transarterial therapy in conjunction with bevacizumab and immunotherapy as a neoadjuvant treatment option for patients with locally advanced HCC. Furthermore, this therapeutic approach is correlated with significant improvements in OS, PFS, and ORR when compared to either direct surgical resection or the combination of bevacizumab and immunotherapy. Notably, the AEs of neoadjuvant therapy were susceptible to effective management and control.

## Data Availability

The original contributions presented in the study are included in the article/**Supplementary Material**. Further inquiries can be directed to the corresponding authors.

## Supplementary material

Supplementary Figure 1 Patient survival was shown by the Kaplan–Meier curves. The OS **(A)** and PFS **(B)** in patients received Neo-surgery with tumor pathological response (pCR vs Non-pCR). OS, overall survival; PFS, progression-free survival; Neo, neoadjuvant; pCR, pathological complete response.

Supplementary Figure 2 Liver function change according to ALT **(A)** and AST **(B)** in patients received TAT-Bev-ICIs, and ALT **(C)** and AST **(D)** in patients received Bev-ICIs. ALT, alanine aminotransferase; AST, aspartate aminotransferase; TAT, transarterial therapy; Bev, bevacizumab; ICIs, immune checkpoint inhibitors.

Supplementary Figure 3 Waterfall plot for tumor size changes of intra-hepatic target lesions. PD, progressive disease; PR, partial response.
