# Supplementary figures and images for: Transarterial therapy combined with bevacizumab plus immune checkpoint inhibitors as a neoadjuvant therapy for locally advanced HCC
Source: Front Immunol. 2024 Dec 23;15:1469302. doi: 10.3389/fimmu.2024.1469302 (PMC11700993; doi:10.3389/fimmu.2024.1469302)

A

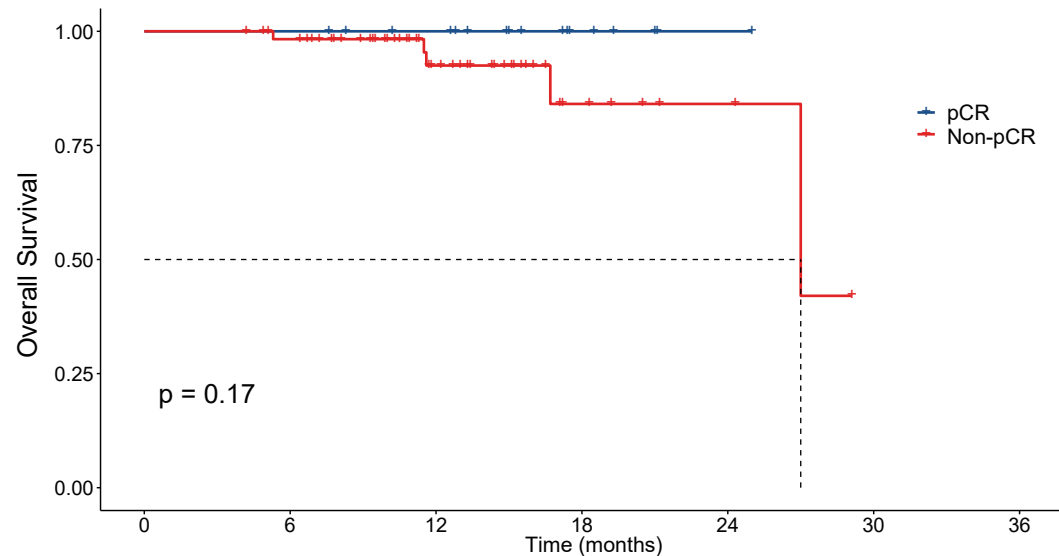

Number at risk

|         |    |    |    |    |    |    |    |
|---------|----|----|----|----|----|----|----|
| pCR     | 18 | 18 | 14 | 5  | 1  | 0  | 0  |
| Non-pCR | 61 | 57 | 29 | 7  | 3  | 0  | 0  |
|         | 0  | 6  | 12 | 18 | 24 | 30 | 36 |

Time (months)

B

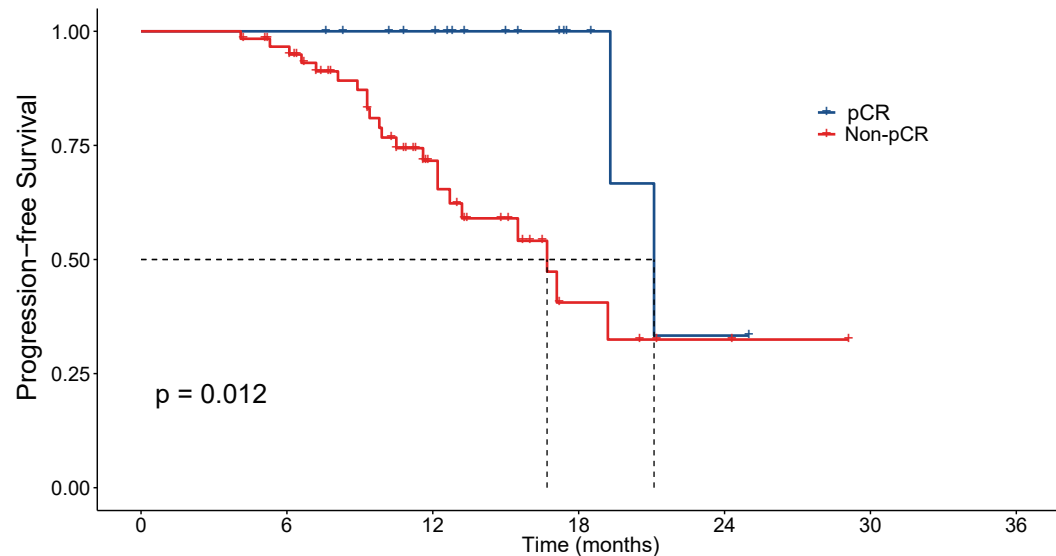

Number at risk

|         |    |    |    |    |    |    |    |
|---------|----|----|----|----|----|----|----|
| pCR     | 18 | 18 | 13 | 4  | 1  | 0  | 0  |
| Non-pCR | 61 | 56 | 23 | 5  | 2  | 0  | 0  |
|         | 0  | 6  | 12 | 18 | 24 | 30 | 36 |

Time (months)

Supplement: Supplementary Figure 1 — Patient survival was shown by the Kaplan–Meier curves. The OS (A) and PFS (B) in patients received Neo-surgery with tumor pathological response (pCR vs Non-pCR). OS, overall survival; PFS, progression-free survival; Neo, neoadjuvant; pCR, pathological complete response. [file DataSheet1.pdf]

A

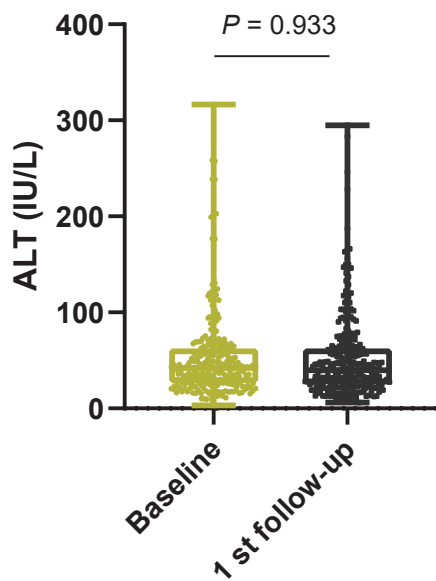

B

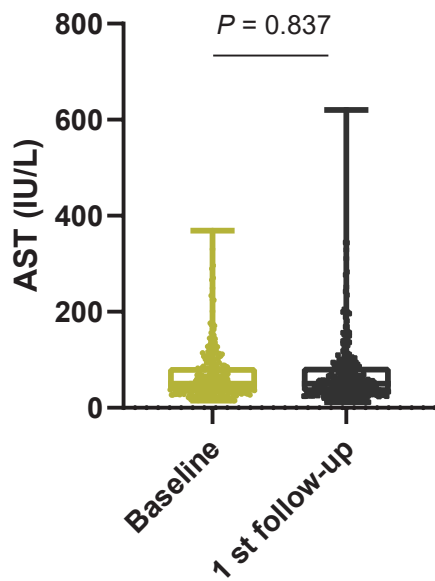

C

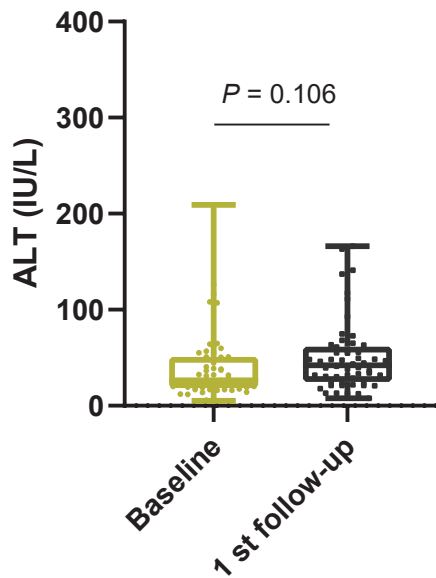

D

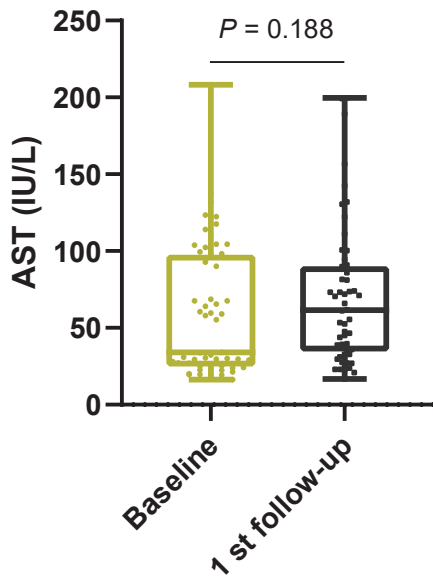

Supplement: Supplementary Figure 2 — Liver function change according to ALT (A) and AST (B) in patients received TAT-Bev-ICIs, and ALT (C) and AST (D) in patients received Bev-ICIs. ALT, alanine aminotransferase; AST, aspartate aminotransferase; TAT, transarterial therapy; Bev, bevacizumab; ICIs, immune checkpoint inhibitors. [file DataSheet2.pdf]

Waterfall plot for Target Lesion Tumor Size

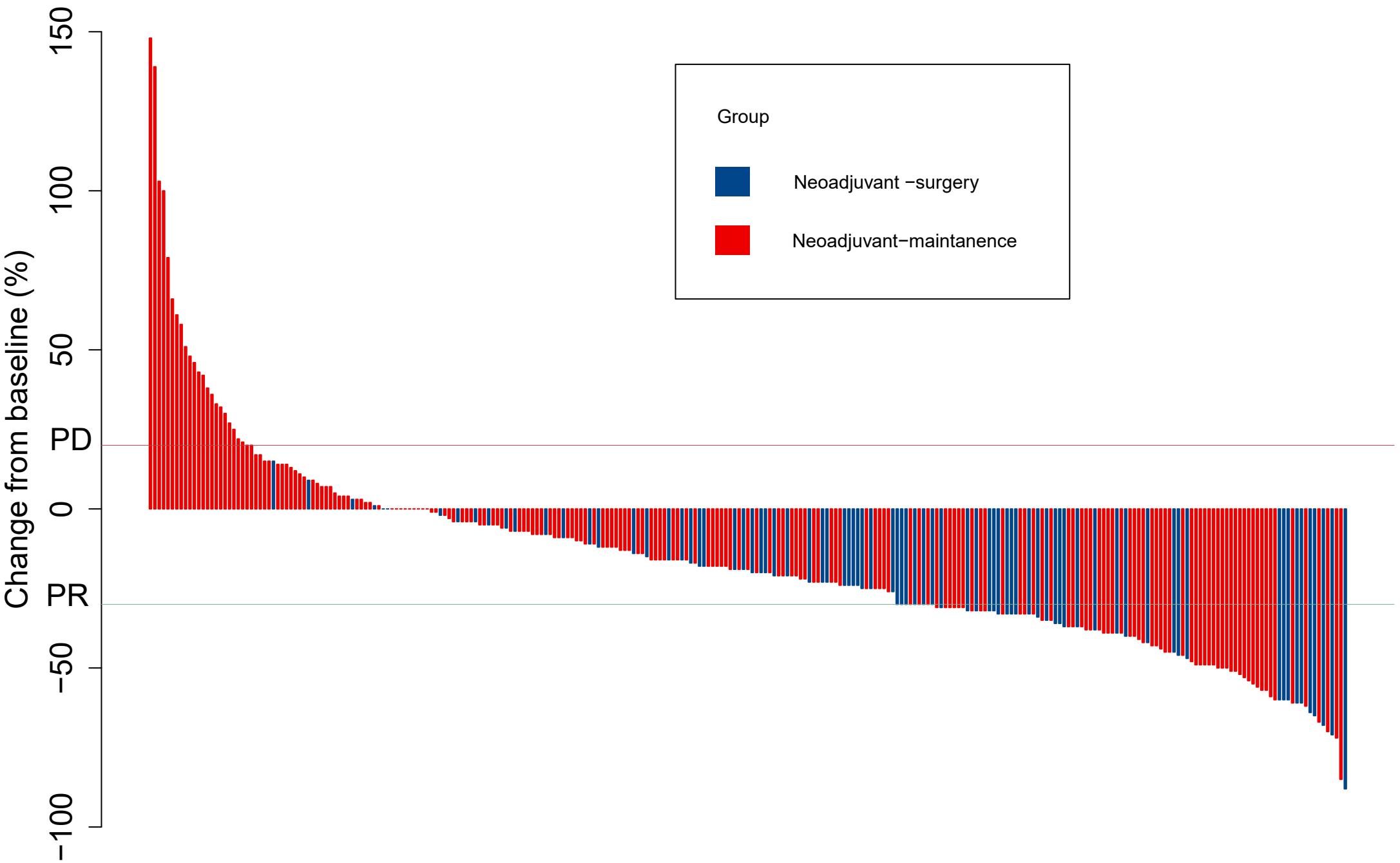

Supplement: Supplementary Figure 3 — Waterfall plot for tumor size changes of intra-hepatic target lesions. PD, progressive disease; PR, partial response. [file DataSheet3.pdf]
