# Supplementary material for: Transarterial therapy combined with bevacizumab plus immune checkpoint inhibitors as a neoadjuvant therapy for locally advanced HCC
Source: Front Immunol. 2024 Dec 23;15:1469302. doi: 10.3389/fimmu.2024.1469302 (PMC11700993; doi:10.3389/fimmu.2024.1469302)
Supplement: Supplementary file 4 [file Table1.docx]

**Table S1: Baseline Characteristics of the 273 Patients Received TAT-Bev-ICIs as Neoadjuvant Therapy before Treatment**

| **Variables** | **TAT-Bev-ICIs (n=273)** |
| --- | --- |
| Age, years | 53.6 (45.4-61.9) |
| Sex |  |
| Male | 242 (88.6) |
| Female | 31 (11.4) |
| Hepatitis infection |  |
| Yes | 236 (86.4) |
| No | 37 (13.6) |
| Liver cirrhosis |  |
| Yes | 170 (62.3) |
| No | 103 (37.7) |
| Preoperative blood tests |  |
| ALT, IU/L | 42.1 (26.6-62.4) |
| AST, IU/L | 50 (33.8-85.3) |
| ALB, g/L | 42.8 (39.6-45.1) |
| TBil, μmol/L | 14.1 (10.7-19.1) |
| AFP, ng/mL | 270 (11.5-13579) |
| WBC, ×10^9^/L | 6.7 (5.4-8.1) |
| HGB, g/L | 144 (131-155) |
| PLT, ×10^9^/L | 203 (146.5-264) |
| PT | 11.9 (11.3-12.7) |
| Largest tumor size, cm | 8.3 (5.3-11.2) |
| Tumor number |  |
| Single | 66 (24.2) |
| Multiple | 207 (75.8) |
| Macrovascular invasion |  |
| Yes | 137 (50.2) |
| No | 136 (49.8) |
| ALBI grade |  |
| I | 198 (72.5) |
| II | 72 (26.4) |
| III | 3 (1.1) |
| BCLC stage |  |
| B | 136 (50.2) |
| C | 137 (49.8) |

**Notes:** Data are presented as median (range), or n (%).

**Abbreviations**: TAT, transarterial therapy; Bev, bevacizumab; ICIs, immune checkpoint inhibitors; AST, aspartate transaminase; ALT, alanine transaminase; ALB, albumin; TBIL, total bilirubin; AFP alpha-fetoprotein; WBC, white blood cell; HGB, hemoglobin; PLT platelet count; PT prothrombin time; ALBI grade, Albumin-Bilirubin grade; BCLC Barcelona clinic liver cancer.
