# Supplementary material for: Transarterial therapy combined with bevacizumab plus immune checkpoint inhibitors as a neoadjuvant therapy for locally advanced HCC
Source: Front Immunol. 2024 Dec 23;15:1469302. doi: 10.3389/fimmu.2024.1469302 (PMC11700993; doi:10.3389/fimmu.2024.1469302)
Supplement: Supplementary file 5 [file Table2.docx]

**Table S2: Baseline Characteristics of the 192 Patients Received Surgery before surgery.**

| **Variables** | **Neo-surgery**  **(n=79)** | **Surgery**  **(n=113)** | ***P* value** |
| --- | --- | --- | --- |
| Largest tumor size, cm | 5.8 (4.4-7.9) | 7.7 (5.5-10) | 0.003 |
| Tumor number |  |  | 0.597 |
| Single | 21 (26.6) | 34 (30.1) |  |
| Multiple | 58 (73.4) | 79 (69.9) |  |
| Macrovascular invasion |  |  | 0.351 |
| Yes | 31 (39.2) | 52 (46) |  |
| No | 48 (60.8) | 61 (54) |  |
| BCLC stage |  |  | 0.351 |
| B | 31 (39.2) | 61 (54) |  |
| C | 48 (60.8) | 52 (46) |  |

**Notes:** Data are presented as median (IQR), or n (%).

**Abbreviations**: BCLC Barcelona clinic liver cancer.
