# Supplementary material for: Transarterial therapy combined with bevacizumab plus immune checkpoint inhibitors as a neoadjuvant therapy for locally advanced HCC
Source: Front Immunol. 2024 Dec 23;15:1469302. doi: 10.3389/fimmu.2024.1469302 (PMC11700993; doi:10.3389/fimmu.2024.1469302)
Supplement: Supplementary file 6 [file Table3.docx]

**Table S3: Objective Treatment-related Adverse Events for Patients Received TAT-Bev-ICIs.**

|  | **Any grade** | **Grade 3-4** |
| --- | --- | --- |
| **Adverse Events** | **TAT-Bev-ICIs**  **(n=273)** | **TAT-Bev-ICIs**  **(n=273)** |
| Rash | 22 (8.1%) | 0 |
| Fever | 109 (39.9%) | 0 |
| Palmar-plantar erythrodysaesthesia syndrome | 38 (13.9%) | 0 |
| Hypothyroidism | 21 (7.7%) | 0 |
| Abdominal pain | 84 (30.8%) | 4 (1.5%) |
| Vomiting | 65 (23.8%) | 5 (1.8%) |
| Diarrhea | 16 (5.9%) | 0 |
| Thrombocytopenia | 20 (7.3%) | 2 (0.7%) |
| Elevated ALT | 98 (35.9%) | 10 (3.7%) |
| Elevated AST | 92 (33.7%) | 11 (4%) |
| Hyperbilirubinemia | 45 (16.5%) | 8 (2.9%) |
| Hypoalbuminemia | 57 (20.9%) | 8 (2.9%) |
| Elevated creatinine | 10 (3.7%) | 0 |
| Sensory neuropathy | 26 (9.2%) | 0 |

**Notes:** Some patients may have multiple immune-related adverse events.

**Abbreviations:** TAT, transarterial therapy; Bev, bevacizumab; ICIs, immune checkpoint inhibitors; ALT, alanine aminotransferase; AST, aspartate aminotransferase.
