# Supplementary material for: Transarterial therapy combined with bevacizumab plus immune checkpoint inhibitors as a neoadjuvant therapy for locally advanced HCC
Source: Front Immunol. 2024 Dec 23;15:1469302. doi: 10.3389/fimmu.2024.1469302 (PMC11700993; doi:10.3389/fimmu.2024.1469302)
Supplement: Supplementary file 7 [file Table4.docx]

**Table S4: Objective Treatment-related Adverse Events for Patients Received Neo-maintenance or Bev-ICIs.**

|  | **Any Grade** | | | **Grade3-4** | | |
| --- | --- | --- | --- | --- | --- | --- |
| **Adverse Events** | **Bev-ICIs (n=54)** | **Neo-maintenance**  **(n=194)** | ***P* value** | **Bev-ICIs (n=54)** | **Neo-maintenance**  **(n=194)** | ***P* value** |
| Rash | 5 (9.3%) | 15 (7.7%) | 0.715 | 0 | 0 | **-** |
| Fever | 5 (9.3%) | 78 (40.2%) | <0.0001 | 0 | 0 | **-** |
| Palmar-plantar erythrodysaesthesia syndrome | 8 (14.8%) | 32 (16.5%) | 0.767 | 0 | 0 | **-** |
| Hypothyroidism | 4 (7.4%) | 15 (7.7%) | 1.000 | 0 | 0 | **-** |
| Abdominal pain | 4 (7.4%) | 55 (28.4%) | 0.003 | 0 | 3 (1.5%) | 1.000 |
| Vomiting | 7 (13%) | 43 (22.2%) | 0.136 | 1 (1.9%) | 5 (2.6%) | 1.000 |
| Diarrhea | 3 (5.6%) | 10 (5.2%) | 1.000 | 0 | 0 | **-** |
| Thrombocytopenia | 4 (7.4%) | 16 (8.2%) | 1.000 | 0 | 2 (1%) | 1.000 |
| Elevated ALT | 10 (18.5%) | 68 (35.1%) | 0.021 | 1 (1.9%) | 8 (4.1%) | 0.705 |
| Elevated AST | 9 (16.7%) | 72 (37.1%) | 0.005 | 1 (1.9%) | 8 (4.1%) | 0.705 |
| Hyperbilirubinemia | 5 (9.3%) | 35 (18%) | 0.121 | 0 | 7 (3.6%) | 0.352 |
| Hypoalbuminemia | 11 (20.4%) | 45 (23.2%) | 0.661 | 2 (3.8%) | 8 (4.1%) | 1.000 |
| Elevated creatinine | 2 (3.7%) | 8 (4.1%) | 1.000 | 0 | 0 | **-** |
| Sensory neuropathy | 3 (5.6%) | 18 (9.3%) | 0.553 | 0 | 0 | **-** |

**Notes:** Some patients may have multiple immune-related adverse events.

**Abbreviations:** Neo, neoadjuvant; Bev, bevacizumab; ICIs, immune checkpoint inhibitors; ALT, alanine aminotransferase; AST, aspartate aminotransferase.
