# Supplementary material for: Transarterial therapy combined with bevacizumab plus immune checkpoint inhibitors as a neoadjuvant therapy for locally advanced HCC
Source: Front Immunol. 2024 Dec 23;15:1469302. doi: 10.3389/fimmu.2024.1469302 (PMC11700993; doi:10.3389/fimmu.2024.1469302)
Supplement: Supplementary file 8 [file Table5.docx]

**Table S5: Best Tumor Responses Evaluated by RECIST1.1 and mRECIST Criteria for Patients Received Neo-maintenance or Bev-ICIs.**

| **Response** | **RECIST1.1** | | | **mRECIST** | | |
| --- | --- | --- | --- | --- | --- | --- |
|  | **Bev-ICIs (n=54)** | **Neo-maintenance**  **(n=194)** | ***P* value** | **Bev-ICIs (n=54)** | **Neo-maintenance**  **(n=194)** | ***P* value** |
| CR | 0 (0) | 2 (1%) | - | 1 (1.9%) | 7 (3.6%) | - |
| PR | 8 (14.8%) | 60 (30.9%) | - | 10 (18.5%) | 64 (33%) | - |
| SD | 26 (48.1%) | 103 (53.1%) | - | 23 (42.6%) | 94 (48.5%) | - |
| PD | 20 (37%) | 29 (14.9%) | - | 20 (37%) | 29 (14.9%) | - |
| ORR | 8 (14.8%) | 62 (31.9%) | 0.013 | 11 (20.4%) | 71 (36.6%) | 0.025 |
| DCR | 34 (63%) | 165 (85.1%) | <0.0001 | 34 (63%) | 165 (85.1%) | <0.0001 |

**Abbreviations:** Neo, neoadjuvant; Bev, bevacizumab; ICIs, immune checkpoint inhibitors; CR, complete response; PR, partial response; SD, stable disease; PD, progressive disease; ORR, objective response rate; DCR, disease control rate.
