# Supplementary material for: Transarterial therapy combined with bevacizumab plus immune checkpoint inhibitors as a neoadjuvant therapy for locally advanced HCC
Source: Front Immunol. 2024 Dec 23;15:1469302. doi: 10.3389/fimmu.2024.1469302 (PMC11700993; doi:10.3389/fimmu.2024.1469302)
Supplement: Supplementary file 9 [file Table6.docx]

**Table S6: Univariate and Multivariate Cox Regression Analyses of Risk Factors for Overall Survival and Progression Free Survival in Patients Received TAT-Bev-ICIs.**

| **Variables** | **OS** | | | | **PFS** | | | |
| --- | --- | --- | --- | --- | --- | --- | --- | --- |
|  | **Univariate** | | **Multivariate** | | **Univariate** | | **Multivariate** | |
|  | **HR(95% CI)** | ***P* value** | **HR(95% CI)** | ***P* value** | **HR(95% CI)** | ***P* value** | **HR(95% CI)** | ***P* value** |
| Age, y (>/≤50) | 0.81 (0.41-1.6） | 0.55 |  |  | 0.8 (0.5-1.28) | 0.36 |  |  |
| Gender(male/female) | 1.08 (0.38-3.08) | 0.88 |  |  | 1.73 (0.75-4) | 0.2 |  |  |
| Hepatitis(yes /no) | 0.68 (0.3-1.56) | 0.36 |  |  | 1.28 (0.61-2.68) | 0.51 |  |  |
| ALB, g/L, (>/≤35) | 1.29 (0.18-9.48) | 0.8 |  |  | 0.4 (0.14-1.13) | 0.083 |  |  |
| TBIL,umol/L,( >/≤17.1) | 1.64 (0.8-3.36) | 0.18 |  |  | 1.23 (0.75-2.03) | 0.41 |  |  |
| AFP,U/mL,(>/≤400) | 2.71 (1.29-5.68) | 0.009 | 2.5 (1.19-5.25) | 0.016 | 1.9 (1.18-3.07) | 0.008 |  |  |
| Largest tumor size (>/≤5 cm) | 2.6 (0.92-7.41) | 0.073 |  |  | 1.15 (0.63-2.1) | 0.66 |  |  |
| Tumor number (>1/1) | 1.98 (0.81-4.84) | 0.14 |  |  | 2.35 (1.2-4.61) | 0.013 | 2.96 (1.45-6.04) | 0.003 |
| Macrovascular invasion (yes/no) | 1.97 (0.97-4.01) | 0.061 |  |  | 1.68 (1.05-2.69) | 0.032 | 1.96 (1.2-3.2) | 0.007 |
| Downstaging liver resection (yes/no) | 0.3 (0.12-0.79) | 0.014 | 0.33 (0.13-0.86) | 0.023 | 0.28 (0.16-0.47) | <0.0001 | 0.29 (0.17-0.5) | <0.0001 |

**Note:** P-value < 0.05 is statistically significant in both univariate and multivariate analyses

**Abbreviations:** TAT, transarterial therapy; Bev, bevacizumab; ICIs, immune checkpoint inhibitors; ALB, albumin; TBIL, total bilirubin; AFP alpha‑fetoprotein.
